# The past and future of USTC: an interview with USTC President Xinhe Bao

**DOI:** 10.1093/nsr/nwy166

**Published:** 2019-02-19

**Authors:** Weijie Zhao, Xiaosu Yi

**Affiliations:** 1 NSR news editor based in Beijing; 2 Li Dak Sum Chair Professor of University of Nottingham Ningbo, China

## Abstract

The University of Science and Technology of China (USTC) is located in Hefei, the capital of Anhui province, and has its own characteristics among the universities in China. Established by the Chinese Academy of Sciences (CAS), USTC is distinctively tinted with a scientific color. It is also famous for its ‘Special Class for the Gifted Young’ and is considered one of the best Chinese universities in the fields of science and technology (S&T). Recently, *National Science Review* interviewed Professor Xinhe Bao, the President of USTC, about the characteristics of the university and the education and research in China. Xinhe Bao is an academician of CAS and has made seminal contributions in catalysis and energy chemistry in the past decades. Before joining USTC, he had worked at Dalian Institute of Chemical Physics (DICP), CAS and Fudan University (Shanghai), and thus possesses an in-depth understanding of the education and research in China.

## UNIQUE CHARACTERISTICS OF USTC

**NSR:** As a university founded by CAS, what are the major characteristics of USTC?

**Bao:** USTC was founded in Beijing in 1958, a crucial time for China. Thus the university was founded with a grand mission: to cultivate S&T talents to meet the needs of the Nation's strategic development particularly in the major S&T projects such as man-made satellites. Therefore, the curriculum was also very unique at that time with the focus on physics, including applied physics, chemical physics and biological physics. Actually, this has been ingrained into the culture of USTC and we are still cultivating talents to meet the needs of the Nation's strategic development. This is the first important characteristic of USTC.

Another characteristic of USTC is its emphasis on the fundamental science courses in its undergraduate program, including mathematics, physics and chemistry. All USTC undergraduate students, regardless of their majors, need to take the course of advanced mathematics for 24 credit hours. Therefore, our undergraduate students are well equipped with basic knowledge. This will make their transitions to other fields smooth, should they decide to change their majors later. This is relatively uncommon in other universities.

The third characteristic is the strong support from CAS, to which USTC belongs. Throughout its 60-year history, the department leaders of USTC are often concurrently served by the directors of the corresponding CAS institutes. I myself served as the director of the Department of Chemical Physics at USTC when I was the director of DICP. Consequently, our students are well informed about the frontier research in the CAS institutes and have the opportunities to join the research of their interest earlier than the students in other universities.

Finally, USTC has been persisting with high-quality education. We enrolled about 1600 undergraduate students in 1958. Now, we enroll about 1860 students each year. Almost all of the Chinese universities have increased the number of students they accept, but we do not. We value the quality of the students we have educated but not the number. Also, we have not built other campuses outside of Hefei except the several research institutes in Suzhou, Shanghai and Beijing. From this point of view, USTC is a university that concentrates on the elite education.

**Figure:**
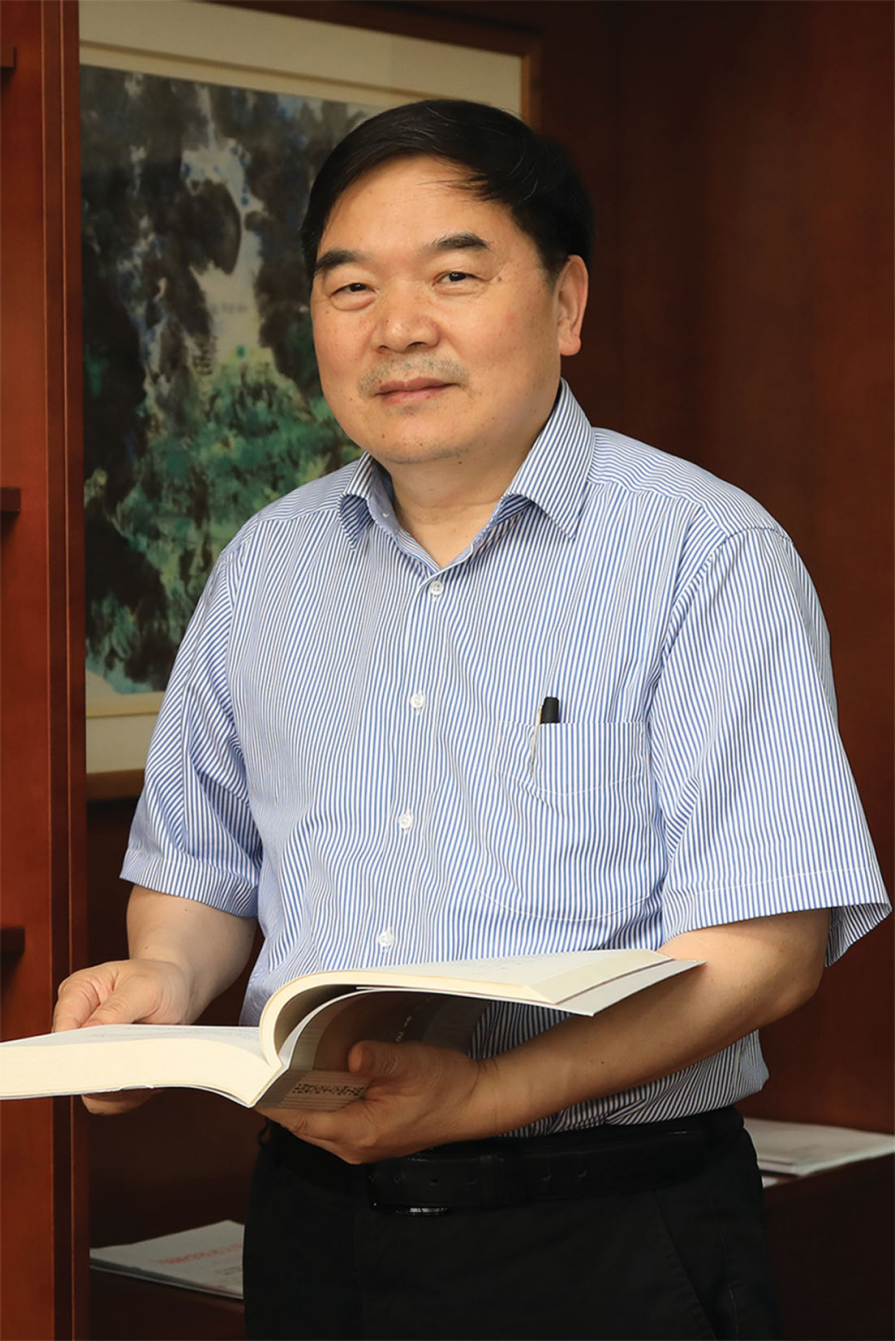
Xinhe Bao, President of USTC and a well-known scientist in catalysis and energy chemistry *(Courtesy of USTC)*.

**NSR:** What are the prospects of USTC graduates? How many of them continue with scientific research after graduation?

**Bao:** About 75% of the USTC undergraduates continue to earn a master’s or doctoral degree after graduation. Most of the remaining 25% graduates join high-tech companies such as Microsoft and Huawei. In addition, there are also many graduates choosing to work in the fields of finance and economy. This is credited to their strong background in mathematics and physics, which they have received at USTC.

**NSR:** What about the study of humanities and social sciences in USTC?

**Bao:** Social science is extremely important for a university. China has been focusing on economic development for a long time, and universities have also been focusing on the cultivation of S&T talents that can immediately contribute to our economic development. Now, we realize that humanistic knowledge is essential for a well-rounded person. The ability of critical thinking, historical vision and aesthetic judgment will also help the innovation in science and technology.

USTC is now strong in some science-related humanities, such as science history, science communication, scientific archaeology, science policy and scientific ethics. We are determined to further develop our humanities and social science programs for our students, and the detailed program will be planned carefully. But we are sure that we will not implement a comprehensive program of all humanities subjects as Peking University and Fudan University. We will continue to focus on the science-related subjects.

All USTC undergraduate students, regardless of their majors, need to take the course of advanced mathematics for 24 credit hours.

—Xinhe Bao

**NSR:** University of Chinese Academy of Sciences (UCAS) began to recruit undergraduate students in 2014 and is developing fast. What are the differences between UCAS and USTC?

**Bao:** These two universities have different missions. The graduate school of USTC, the predecessor of UCAS, was established in 1978. Before 2000, the institutes of CAS had individually recruited and trained their graduate students. In 2000, the graduate school of USTC was reorganized into the graduate school of CAS and the management of all CAS graduate students became unified there. Several years later, some problems with that system were recognized. For example, students and their professors were not considered part of the national education system and could not enjoy the preferential policies provided for students and teachers. Thus, CAS decided to reorganize the graduate school again into a university in 2014, which was named University of Chinese Academy of Sciences by the then Chinese Vice Premier Liu Yandong.

UCAS was established for the purpose of the education of post-graduate students, which is still its main mission. Considering that undergraduate education is an integral part of a university, UCAS started to enroll undergraduates in 2014. However, the scale of its undergraduate education is relatively small, with only about 400 students enrolled every year. In contrast, the education of undergraduates and that of post-graduates are equally well developed in USTC.

Personally, I believe that CAS can benefit from this binary education system. The coexistence and inter-support of USTC and UCAS are favorable for the education of young talents. We value each other as close partners with complimentary features. We are actively communicating with UCAS to promote collaboration in student enrollment and the mutual recognition of undergraduate degrees. I sincerely wish and I am also confident that UCAS will become stronger and stronger in all aspects.

## SPECIAL CLASS FOR THE GIFTED YOUNG

**NSR:** The Special Class for the Gifted Young (SCGY) is one of the distinguished signature of USTC. It has cultivated many outstanding talents throughout its 40-year history. What is the current status of SCGY?

**Bao:** The birth of SCGY can be traced back to 1977, when a college teacher in Jiangxi Province wrote a letter to CAS requesting special education to an extremely gifted 12-year-old boy. As a university of CAS, USTC sent representatives to Jiangxi to assess the intelligence of this boy and several other children. They found that some of them were indeed outstanding. Therefore, with the support of the Chinese Vice Premier Fang Yi and CAS president Moruo Guo, SCGY was established in 1978. The establishment of SCGY sparked great excitement throughout China and USTC became widely known and highly respected.

SCGY enrolled 21 students in 1978. Now we have about 50 students a year. To be qualified, these students have to be younger than 16 years old and have to score above the cut-off line of the first class at the national college entrance examination.

Later on, we established another special group called the Innovation Class in 1985, which enrolled talented students from the second grade of high school. Every year, we receive about 20 000 applications and select about 5000 of them who are allowed to take part in the national college entrance examination. Only those who pass the examinations and interviews are enrolled into this class, usually less than 200 students.

Students of both classes are currently organized into our School of Gifted Young (also abbreviated as SCGY). SCGY has cultivated many outstanding graduates, such as academicians of the US National Academy of Sciences including Xiaowei Zhuang and Liqun Luo, academicians of CAS including Jiangfeng Du, as well as outstanding talents in the non-scientific fields including the President of Baidu Yaqin Zhang.

**NSR:** What are the special features in the education of SCGY students compared to regular students?

**Bao:** SCGY students are usually younger and less mature than regular students, so we implement special measures for their education. For example, SCGY students do not belong to certain departments, and can decide on their major in the third or fourth year. Their courses are also specially designed. Furthermore, we encourage SCGY students to get a second bachelor degree. Since they enter the university at a relatively young age, they can freely choose to complete two degrees within 5 years.

**Figure:**
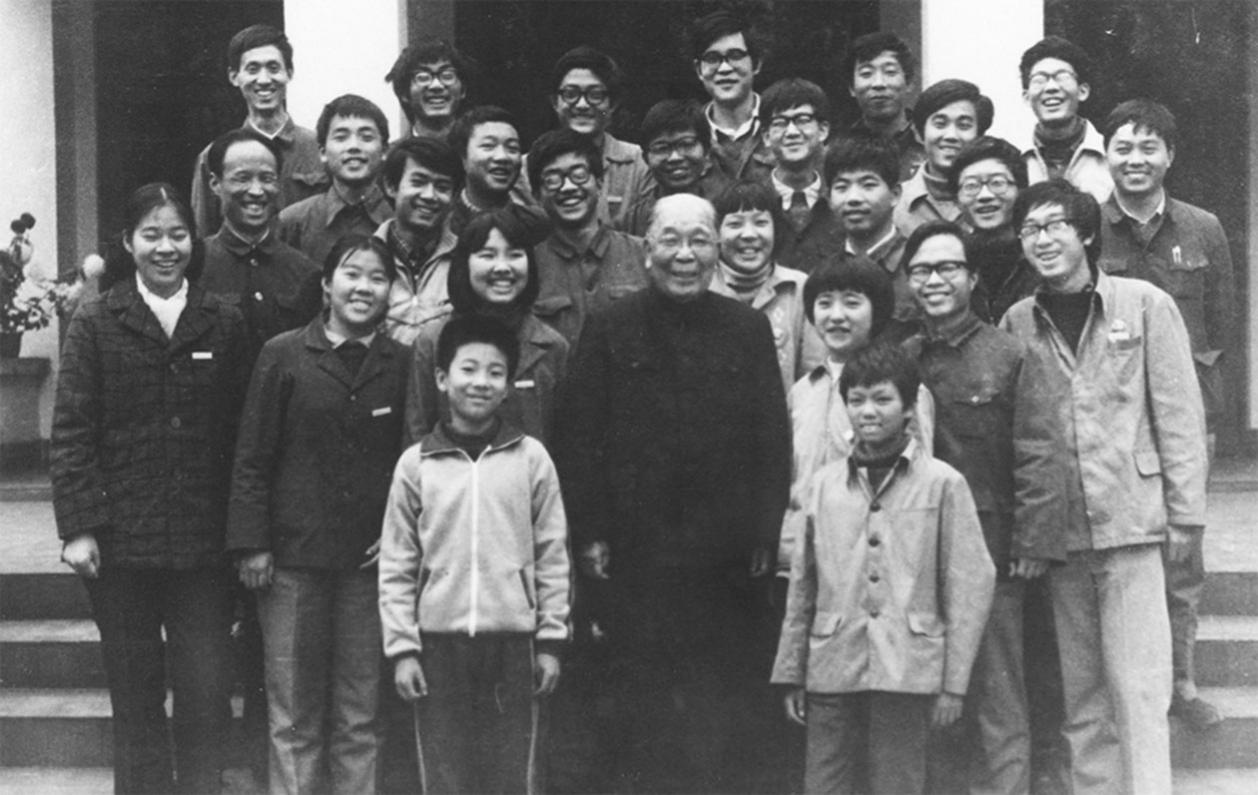
SCGY students with the then-President of USTC, Jici Yan, pictured in 1981 *(Courtesy of USTC)*.

**NSR:** Are there special classes similar to SCGY in other universities?

**Bao:** Not long after the establishment of SCGY, more than 20 Chinese universities, including Peking University and Tsinghua University, started their own special classes for young gifted students. However, USTC and Xi’an Jiaotong University (XJTLU) are perhaps the only two that have kept the special classes until today.

Moreover, the XJTLU special class is different from ours. They recruit students from the junior middle school and the selected students, although not having to take the national examination, will need to complete their high-school courses before entering XJTLU. In that sense, their special class is more like a preparatory class. In contrast, our SCGY students enter the university education system as soon as they are enrolled.

Furthermore, the ‘Training Program of Top-level Students in Fundamental Sciences’ proposed by the Chinese Ministry of Education as well as many special classes in various universities partly take the SCGY as a reference. In this sense, SCGY has made valuable contribution to the reform of Chinese education system.

**NSR:** What are the plans for the future development of USTC?

**Bao:** Recently, President Xi Jinping has proposed five key missions for Chinese universities: talent cultivation, scientific research, community service, cultural heritage and international collaboration.

As a university, talent cultivation is our priority. We will continue to improve the quality of undergraduate and post-graduate education, to provide better general education and specialized eduction on innovation and entrepreneurship by various reforms.

As a part of CAS, we also emphasize the importance of scientific research. In recent years, USTC has made many significant achievements in the fields of quantum communication, quantum computing, artificial intelligence (AI), cyberspace security, engineering and medical science. Take the field of AI as an example, many newly established Chinese AI companies, including iFlytek, Sense Time, Unisound and Cambricon, were established by USTC graduates. In the future, USTC will continue to strengthen the efforts in both research and eduction, in order to advance the S&T frontiers to address the major needs of the Nation's development.

## REGIONAL IMBALANCE OF RESEARCH AND EDUCATION

**NSR:** It is said that USTC is localized and dominated by Anhui people. Is that true?

**Bao:** I think localization to a certain extent is normal for all universities. USTC is not particularly localized compared with other universities.

For student enrollment, less than one-sixth of USTC undergraduate students come from Anhui. This proportion is lower than that of Shanghai students in Fudan University and that of Zhejiang students in Zhejiang University.

Meanwhile, we do have a relatively high proportion of Anhui teachers. This is again a normal outcome from the process of talent recruitment as scholars from Anhui and the alumni are more interested in joining USTC. What matters is not where the teachers come from, but the quality of their teaching and researching.

**NSR:** Hefei is not one of the most developed cities. Is the location a limitation for the development of USTC?

**Bao:** Yes, there are certain limitations. It is reasonable that students and professors prefer to study or work in better developed cities. The commonly considered top universities in China, referred to as ‘TPEC5’, include Tsinghua University, Peking University and five other universities in East China (Fudan University, Shanghai Jiaotong University, Nanjing University, Zhejiang University and USTC). All these universities except USTC are located in major cities.

Under these circumstances, USTC is still able to continuously recruit talented teachers and students and nurture high-level researchers despite its location in Hefei. This can be ascribed to its strength and influence that has gained over the years.

In this sense, it is similar to DICP. DICP develops very well and is a world leading research institute in the field of catalysis although it is located in the Northeast of China where it is not the most developed city. I think there are two reasons. Firstly, even though Hefei and Dalian are not metropolises, their geographical locations and urban development are sufficiently good to support a satisfactory lifestyle. Secondly, both USTC and DICP are strong in research and offer a very good cultural atmosphere for researchers and teachers. On top of that, both provide rather good salaries and benefits.

Where does the unfairness come from? The answer lies in the inequality in primary-school and middle-school education.

—Xinhe Bao

**NSR:** From another point of view, does USTC also facilitate the development of Hefei?

**Bao:** Indeed, USTC and Hefei are tightly connected. USTC has greatly enhanced the development of Hefei's S&T industry and the impact of Hefei. Actually, many foreign researchers started to learn about Hefei from USTC. USTC must be acknowledged for the presence of many large-scale scientific facilities, research institutes and S&T companies in Hefei. Moreover, a number of USTC graduates have settled in Hefei, which may further facilitate its development.

**NSR:** What are the reasons for this regional imbalance?

**Bao:** I think this is a natural consequence of China's current development which is not perfectly balanced. If all of the cities become well developed in the future, or the transport system becomes affordable and convenient enough that we can reach the major cities within a couple of hours, this regional imbalance of universities and research institutions will naturally vanish. Such regional imbalance, although more modest, also exists in the USA.

**NSR:** Besides the regional imbalance of institutions, there is concern about the uneven allocation of educational resources: children from poor families have increasing difficulties to enter high-level universities. What can universities do to promote equity in education?

**Bao:** This is an important issue. Since China's reform and opening up, the national college entrance examination system has been implemented, which is considered to be rather fair. Many, including myself, have benefited from this system. The examination and college enrollment process are both considered fair, but where does the unfairness come from? The answer lies in the inequality in primary-school and middle-school education.

Nowadays, many middle-school students from well-off families are extremely well-educated. They begin to learn about natural science and social science from a very young age and acquire knowledge that is inaccessible by children in poorer families. If children cannot receive basic education from the beginning, it is more difficult for them to perform well in the national examination and enter the university.

To improve equality, the government has proposed the Special Enrollment Plan for Poverty-Stricken Areas. According to this plan, each university is required to enroll a certain number of students from poverty-stricken provinces such as Guizhou, Xinjiang and Yunnan with a relatively low cut-off score. In USTC, we enroll about 200 students through this plan each year. We also have special policies for rural students.

Within the university, all students are treated equally. Special support is available to the less well-off students, including scholarships, grants and special plans that encourage and support them to study abroad. Furthermore, we try to minimize the impact of the financial status in the campus, to ensure the mental health of the less well-off students. We discourage students to flaunt their wealth and no one can enjoy better accommodation by paying more money.

Both our government and universities pay great attention to the inequality in education and have made many regulations. However, I think that to completely banish such inequality, much more and wider efforts are needed from the whole society.

## ENERGY CHEMISTRY RESEARCH IN CHINA

**NSR:** What are the characteristics of China's energy system?

**Bao:** I have talked about this topic in many different occasions. Although we can learn from the developed countries in many realms of sciences and technologies, we cannot follow their energy development strategies because our structure of the energy resources is completely different from theirs. For example, the USA has basically achieved energy self-sufficiency largely by increasing production of unconventional fossil energy such as shale gas and shale oil. The German energy system is currently largely relying on renewable resources such as wind energy and solar energy. In China, we do not have abundant exploitable shale gas resources so far; and, as a country with a large energy demand, we cannot merely rely on renewable resources. We have to develop our own energy strategy based upon our own energy structure.

Generally speaking, China is short of energy resources, especially liquid energy resources. We consume around 500 million tons crude oil each year, but our total annual oil production is only around 200 million tons. It means that 65% of the oil we use comes from import. We are also short of natural gas. Many cities are transforming their winter central heating systems from coal- to gas-powered. Much of the gas used here is imported from Russia and the Middle East. The only energy resource that does not need import is coal, which occupies about 65% of China's energy consumption, whereas all the renewable energy resources add up to less than 15%. It is widely recognized that coal will still be the dominating energy resource in our energy structure in the foreseeable future.

We have to develop our own energy strategy based upon our own energy structure.

—Xinhe Bao

Unfortunately, the burning of coal causes many problems. It produces carbon dioxide which contributes to the greenhouse effect. It also produces many other pollutants that lead to acid rain and haze. How to cleanly and effectively use our coal resources is an extremely important scientific issue.

**NSR:** What are your group's major contributions to this field?

**Bao:** I have been working on catalysis and energy chemistry for more than 35 years since my post-graduate study. Catalysis is a key pillar of the energy industry and my main research involves optimized utilization of natural gas and coal. We developed a new catalyst and catalytic reaction converting methane (the main component of natural gas) directly to high-value chemicals within one step without carbon-dioxide emission. In the field of coal research, we developed a novel technology of transforming syngas, which is the product of coal gasification, into chemicals such as light olefins within one step with less water usage. Carbon-dioxide emission and water usage are the key problems confronting the coal industry. Successful reduction of carbon emission and water usage is of significance for the coal industry. These two pieces of work, both published in *Science*, are highly impactful and were awarded as one of the top 10 science advances in China in 2014 and 2016, respectively. Now, we are collaborating with an applied research team and industrial partners trying to turn these scientific findings into real technologies.

In recent years, I have been working in the universities in Shanghai and Hefei, but my research group remains in DICP, Dalian. I started from fundamental research, and will continue to focus on fundamental research on catalysis and the development and applications of new catalysts. Catalysis has a history as long as 150 years, but there were few revolutionary breakthroughs in the field of fundamental research. It is still considered as a black box concerning the reaction mechanisms. In my opinion, there may have been some epistemological or methodological limitations. We may have to jump out of the conventional ideas and reconsider the fundamentals of catalysis. Recently, we are trying to solve some of the key fundamental problems in this field and have gained some understanding that are recognized by international peers.

I will be very happy if I can make contributions to the development of catalysis fundamentals and turn the two aforementioned scientific findings into real technologies within my lifetime.

**NSR:** How do you balance your research in Dalian and education at Hefei? Both of them are demanding.

**Bao:** Actually, most of the senior professors are extremely busy. There are too many meetings to attend and too many issues to deal with. We have little time to work in the lab and to have face-to-face discussions with students, which I think is a great pity.

I am extremely lucky that my research group in DICP is already well developed. There are a number of talented young researchers, including several principal investigators who are strong and capable to train students and advance our research work simultaneously. They are doing very well and we only need to discuss together when it is needed. The pity I have been carrying all the time is that there is not much time for discussion with the students. I feel sorry for that.

**NSR:** What advice have you got for young researchers?

**Bao:** For young professors, I would advise them to closely work with the students. One should not just sit in the office and act as a boss once he or she get a professorship position. The only way to significant discoveries, sometimes from unusual observations, is to work and discuss with the students in the lab, and to elevate the students’ work with your knowledge of science. This is also my requirement for young researchers in my own team.

For students, my advice is to keep curiosity and do what you are really interested in. If you are not interested in your research, but only work for the sake of a master’s or doctoral degree, you will be unable to resonate with your work and will not be passionate enough to try every possibility to push forward the work. Besides, you should always be critical. Only by critically appraising the existing papers can you raise and solve new questions and make innovative discoveries.

